# Pre-educational intervention survey of healthcare practioners’ compliance with infection prevention measures in cardiothoracic surgery: low compliance but internationally comparable surgical site infection rate

**DOI:** 10.1186/1753-6561-5-S6-O59

**Published:** 2011-06-29

**Authors:** E Tartari, J Mamo, M Borg

**Affiliations:** 1 Infection Control Unit, Mater dei Hospital, Msida, Malta; 2 Public Health Department, Mater dei Hospital, Msida, Malta

## Introduction / objectives

Surgical site infections (SSI) are challenging problems leading to significant postoperative morbidity and mortality and may reflect the level of adherence to infection control policies.

## Methods

We used a structured observational method to collect data about infection control practices amongst surgeons, anaesthetists, nurses, cardiopulmonary bypass technicians and orderlies practicing in the cardiac operating room during open-heart surgery at Mater Dei Hospital. To prevent bias, we did not disclose the actual procedures observed to the surgical team members, who however knew they were being observed for infection control practices. We measured the 30-day SSI rate by post-discharge telephonic surveillance amongst surviving open-heart surgery patients who had consented to the survey.

## Results

We observed infection control practices during 30 randomly chosen operations and found higher level of inadequate practices related to environmental disinfection, hand hygiene, operating room traffic and surgical attire of non-scrubbed personnel (anaesthesiologists and cardiopulmonary bypass technicians).

140 of 155 patients who underwent open-heart surgery were followed up, achieving a response rate of 91.5%. Superficial and deep surgical site infections rates were 16.4% and 4.3% respectively, including both sternal and harvest-site infections.

## Conclusion

We found poor compliance with infection control practices of non-scrubbed personnel involved in cardiac surgery and observed a high surgical site infection rate, the majority being leg wound infections following saphenous vein harvesting.”

Keywords ‘Operating room practices’, ‘cardiac surgery’, ‘surgical site infections’.

## Disclosure of interest

None declared.

